# Evaluating the Structural and Construct Validity of the Pain Understanding and Confidence Questionnaire for Assessing Physical Therapists’ Pain Management Competence: A Cross-Sectional Study

**DOI:** 10.7759/cureus.66347

**Published:** 2024-08-07

**Authors:** Hiroshi Takasaki

**Affiliations:** 1 Department of Physical Therapy, Saitama Prefectural University, Koshigaya, JPN

**Keywords:** whiplash, pain neuroscience education, pain management, knowledge, chronic pain, unidimensionality

## Abstract

Background

The Pain Understanding and Confidence Questionnaire (PUnCQ) comprises two parts: the first assesses clinical judgments based on contemporary pain knowledge, and the second consists of items querying confidence in pain management for the presented vignette. In contrast to existing measures, PUnCQ can evaluate a therapist’s capacity to make appropriate clinical decisions within a specific vignette. Thus, PUnCQ may be a promising measure to assess the clinical competence of physical therapists in pain management. This study evaluated the structural and construct validity of PUnCQ.

Methodology

Eligible participants were two cohorts of physical therapists managing patients with pain. PUnCQ and Knowledge and Attitudes of Pain (KNAP) data were collected using an anonymous survey. Confirmatory factor analysis was conducted for both parts of the PUnCQ, and an exploratory factor analysis was conducted when multidimensionality was suspected. Construct validity was assessed with the hypothesis that Pearson’s r values to KNAP scores, indicating knowledge about modern pain science and biopsychosocial attitudes toward pain, were expected to be 0.3-0.5 in part one and >0.5 in part two.

Results

Data from 112 participants were analyzed. PUnCQ part one fully satisfied the predetermined criteria for unidimensionality, but part two did not. Exploratory factor analysis for part two revealed a two-factor structure: a 14-item Factor 1 labeled “pain management” and a seven-item Factor 2 labeled “medication guidance and pain mechanism,” while Cronbach’s alpha was 0.98 across all items. Statistically significant correlations were detected with the KNAP in each part of the PUnCQ (r = 0.26 in part one and r = 0.41 in part two).

Conclusion

PUnCQ has structural validity and an aspect of construct validity.

## Introduction

Pain is the main reason patients seek musculoskeletal care. A comprehensive understanding of pain is essential for patient education, which is a core component of optimal musculoskeletal management [1] and whose effective administration is a recent research priority [2]. Research has focused on the attitudes and knowledge of physical therapists regarding pain, resulting in the development of assessment methods. For example, the Pain Attitudes and Beliefs Scale for Physiotherapists [3] evaluates the balance between the biological perspective, considering the extent of tissue damage in low back pain, and biopsychosocial factors that can exacerbate pain beyond tissue damage. Similarly, the Neurophysiology of Pain Questionnaire [4,5] was developed and modified to measure knowledge of neurophysiological pain mechanisms. While these measures contributed to evidence-based practice [6], the recent recognition of the effectiveness of pain neuroscience education [7,8] has led to the development of the Knowledge and Attitudes of Pain (KNAP), which assesses knowledge about modern pain science and biopsychosocial attitudes required for health care professionals [9]. However, these existing measures primarily assess general knowledge and attitudes, although clinical decision-making should be made by considering the patient’s biopsychosocial status, medical history, thoughts, and preferences as a whole. Therefore, they lack an evaluation of their ability to perform appropriate pain management in real clinical settings.

In response to these limitations, the Pain Understanding and Confidence Questionnaire (PUnCQ), introduced in 2020 [10,11], presents a clinical vignette featuring whiplash-associated disorders. PUnCQ probes 12 aspects of pain management. In contrast to the existing measures, PUnCQ surpasses general pain knowledge assessment, focusing on the competence of evidence-based clinical decision-making within a specific vignette. This unique approach positions PUnCQ as a promising tool for evaluating an aspect of the clinical competence of physical therapists in pain management. The PUnCQ has two parts: the first assesses the clinical competence of clinical decision-making based on contemporary knowledge of pain, and the second consists of 21 items querying confidence in pain management for the vignette. PUnCQ reflects the core curriculum of the International Association for the Study of Pain [12] and the British Pain Society [13]. Moreover, PUnCQ was developed by an expert panel including an anesthetist, a nurse, a pharmacist, a physical therapist, a psychologist, and an occupational therapist, indicating content validity. A previous study [10] investigated internal consistency for both parts among 133 undergraduate students and reported Cronbach’s alpha values of 0.58 for part one and 0.96 for part two. However, validation through factor analysis for construct validity, recommended by the COnsensus-based Standards for the selection of health Measurement INstruments (COSMIN) initiative [14], has not been conducted. Therefore, PUnCQ, while showing some evidence of internal structure, requires further construct validity using factor analysis.

When evaluating internal structure, internal consistency is an important but insufficient indicator, and unidimensionality is crucial for using a representative score for a certain concept. Despite content validity and satisfactory internal consistency across all items, a lack of unidimensionality may require scoring adjustments, such as summing averaged scores in each subscale to reach an overall score.

This study had two primary objectives: to evaluate the structural validity of the PUnCQ by assessing the unidimensionality of its two parts through factor analysis and to assess the construct validity of the PUnCQ by testing its correlation with the KNAP. These objectives aim to provide evidence for the validity of the PUnCQ as a tool for assessing pain management competence in physical therapists.

## Materials and methods

Design

This cross-sectional study involved the data analysis of an anonymous paper-based survey administered to members of the Japanese Society of Allied Health and Rehabilitation and an anonymous online survey administered to members of the Japan Branch of the International McKenzie Institute. This study was an anonymous survey, and by completing the survey form, participants were exempted from submitting a written consent form for this study. The Ethics Committee of the Saitama Prefectural University approved the study (approval number 23100).

Participants

Following COSMIN guidelines [14], the sample size calculation indicated at least 105 participants to conduct a confirmatory factor analysis of the 21-item PUnCQ. Surveys were conducted among members of the Japanese Society of Allied Health and Rehabilitation (n = 263) and the Japan Branch of the International McKenzie Institute (n = 387), assuming a survey participation of approximately 20% based on previous studies [15-19].

The inclusion criterion was individuals registered as members of the respective organizations. Members of the Japan Branch of the International McKenzie Institute with registered email addresses were targeted.

The exclusion criteria were individuals not currently involved in treating patients with pain, those from professions other than physical therapy, and those with missing data. Duplicates across the two cohorts were excluded. The data collection period for each organization was two weeks.

Variables

The PUnCQ utilizes a clinical vignette featuring a whiplash-associated disorder. In part one, participants respond to 12 clinical judgments related to pain. Part two assesses confidence in 21 management skills. In part one, each of the five choices per clinical judgment is assigned a score of 1 for a correct response. Inappropriate choices, including “do not know,” receive a score of 0. The total score ranges from 0 to 12. In part two, participants rate their confidence in each management skill on an 11-point scale from 0 (not at all confident) to 10 (no problem). The mean score ranges from 0 to 10. This study used the Japanese PUnCQ [20].

The KNAP comprises 30 items that assess the participants’ knowledge of modern pain science and biopsychosocial attitudes toward pain. Respondents rate their agreement with statements on a 6-point scale. Following COSMIN guidelines, the KNAP underwent content validity verification [9]. Structural validity was assessed through exploratory factor and Rasch analysis, using data from 424 students in a four-year entry-level physical therapy education program [9]. In addition to internal consistency, hypothesis testing, test-retest reliability, and responsiveness were examined [9]. The internal consistency of KNAP, measured by Cronbach’s alpha, was 0.8 [9]. Scores were weighted for each item [9], resulting in a total score range of 0 to 150, where higher scores indicate greater knowledge of modern pain science and biopsychosocial attitudes toward pain. This study used the Japanese KNAP [21].

Analysis

IBM®SPSS® Amos™ 20.0 (IBM Corp., Armonk, NY, USA) was used for the confirmatory factor analysis. IBM SPSS Statistics for Windows, Version 28.0 (Released 2021; IBM Corp.) was used for other statistical analyses. The significance level was 5%.

Structural Validity

Unidimensionality was assumed when one of the following criteria was satisfied: (1) chi-squared/degree of freedom <3; (2) comparative fit of index >0.95; (3) goodness-of-fit index >0.90; (4) adjusted goodness-of-fit index >0.85; (5) root mean square error of approximation <0.06; or (6) standardized root mean square residual <0.08 [14,22-24]. However, for the conservatory assessment of structural validity, when one of the criteria was not satisfied, exploratory factor analysis was conducted with the maximum likelihood method and direct oblimin rotation as recommended in a previous study [25]. Factor solutions with eigenvalues >1 were investigated. Additionally, the Kaiser-Meyer-Olkin measure was calculated, and Bartlett’s sphericity test was conducted with the acceptance criteria of a Kaiser-Meyer-Olkin measure >0.5 and a p-value <0.05. Subsequently, Cronbach’s alpha was calculated for each factor identified in the exploratory factor analysis and across all items. Acceptable internal consistency was deemed an alpha value ≥0.7 [14].

Hypothesis Testing for Construct Validity

Each part of the PUnCQ was expected to have a statistically significant correlation to KNAP. Furthermore, part two of the PUnCQ uses an 11-point numerical confidence rating scale, which was expected to be similar to KNAP. Therefore, Pearson’s r correlation to KNAP was expected to be >0.5 [14]. In contrast, part two of the PUnCQ uses binary variables and indicates the competence of evidence-based clinical decision-making within a specific vignette, which was expected to be dissimilar to KNAP. Therefore, Pearson’s r correlation to KNAP was expected to be 0.3-0.5 [14].

## Results

Responses were obtained from 78 individuals (29.7%) in the Japanese Society of Allied Health and Rehabilitation cohort and 80 (20.1%) in the Japan Branch of the International McKenzie Institute cohort. Eighteen individuals in the Japanese Society of Allied Health and Rehabilitation cohort were excluded, yielding 60 valid responses. Similarly, 28 individuals from the Japan branch of the International McKenzie Institute cohort were excluded, yielding 52 valid responses. In total, there were 112 valid responses. The summary of participant demographics is presented in Table 1.

**Table 1:** Summary of the 112 participants

Variables	N = 112
Age (years), mean (SD)	36.7 (8.7)
Clinical experience (years), mean (SD)	12.5 (7.5)
Sex	
n of men (%)	89 (79.5%)
n of women (%)	23 (20.5%)
Academic degree	
n of career college (%)	48 (42.9%)
n of junior college or college (%)	52 (46.4%)
n of master’s degree (%)	10 (8.9%)
n of doctoral degree (%)	2 (1.8%)

**Figure 1:**
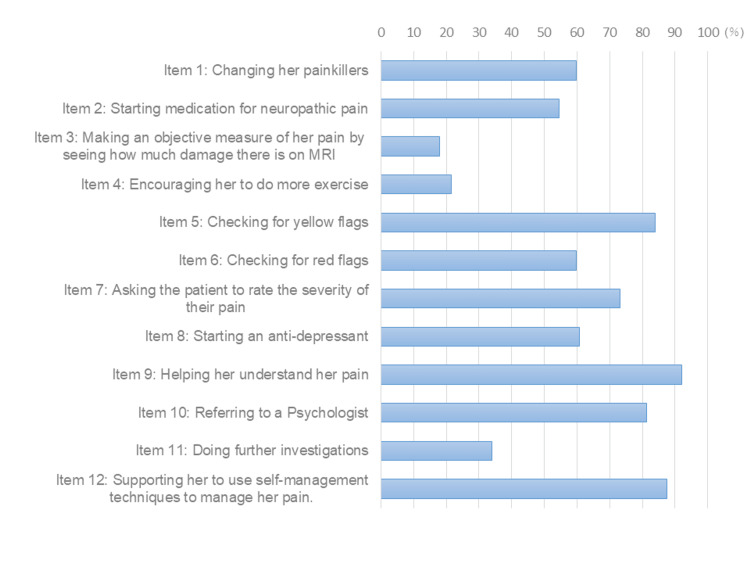
Response distributions of the 112 participants in part one of the PUnCQ PUnCQ, Pain Understanding and Confidence Questionnaire

For the 112 participants, the mean (SD) for part one of the PUnCQ was 7.3 (1.6), and correct response rates are presented for each item in Figure 1. In particular, items 3, 4, and 11 had a correct response rate of less than 40%. The mean (SD) for part two of the PUnCQ was 5.2 (2.0). The mean (SD) for KNAP was 81.1 (6.3).

The results of the confirmatory factor analysis are summarized in Table 2. Part one of the PUnCQ met all six criteria for unidimensionality, while part two met only one. Consequently, an exploratory factor analysis was conducted for part two. The results revealed two-factor structures (Table 3) (Kaiser-Meyer-Olkin measure = 0.933 and p < 0.001 in Bartlett’s sphericity test). The first factor, comprising 14 items, was labeled “pain management,” and the second factor, comprising seven items, “medication guidance and pain mechanism.” Cronbach’s alpha for the first and second factors and all items was 0.98, 0.93, and 0.98, respectively, indicating acceptable internal consistency.

**Table 2:** Results of the confirmatory factor analysis * Satisfied with the predetermined unidimensionality criteria ADFI, adjusted goodness-of-fit index; CFI, comparative fit of index; CMIN/df, chi-squared/degree of freedom; GFI, goodness-of-fit index; PUnCQ, Pain Understanding and Confidence Questionnaire; RMSEA, root mean square error of approximation; SRMR, standardized root mean square residual

PUnCQ	CMIN/df	CFI	GFI	ADFI	RMSEA	SRMR
Part 1	0.868*	1.000*	0.935*	0.906*	<0.001*	0.070*
Part 2	4.516	0.778	0.545	0.444	0.178	0.068*

**Table 3:** Factor loading in part two of the PUnCQ PUnCQ, Pain Understanding and Confidence Questionnaire

Item description	Factor 1	Factor 2
Be able to help her understand that psychological factors influence an individual’s pain experience and pain behaviors in both acute and chronic pain.	1.043	-0.158
Be able to help her understand that social factors influence an individual’s pain experience and pain behaviors in both acute and chronic pain.	1.012	-0.107
In the absence of a clear diagnosis, provide useful information in a way that is helpful from her perspective and will allow her to move toward self-management.	1	-0.089
Be able to help her understand that biological factors influence an individual’s pain experience and pain behaviors in both acute and chronic pain.	1	-0.088
Signpost her with suitable information.	0.843	0.071
Explain the role of psychological interventions in pain management.	0.825	0.11
Know whether you should refer her for more specialist assessment.	0.783	0.035
Assess her understanding of pain and identify fears that may act as barriers to effective management.	0.705	0.186
Describe the role of pacing and activity management.	0.687	0.167
Discuss where self-management is a priority and should be encouraged and supported.	0.675	0.254
Conduct a comprehensive assessment of pain using valid and reliable tools.	0.629	0.187
List the common red flags associated with pain.	0.529	0.349
Describe the contribution of biological, psychological, and social factors to individual variation in pain perception, behavior, and expression.	0.49	0.445
Describe yellow flags and discuss their impact on the management of pain.	0.485	0.436
Differentiate between physical dependence, substance use disorder, misuse, tolerance, addiction, and nonadherence.	-0.062	0.859
Explain the strengths and weaknesses of the World Health Organization analgesic ladder.	-0.064	0.78
Explain the principles of gate control theory and the concepts of pain modulation and neuroplasticity.	0.058	0.703
Explain the main structures involved in pain and how they interact in the pain experience.	0.224	0.701
Diagnose the differences between nociceptive pain and neuropathic pain.	0.227	0.639
Explain analgesic effects, side effects, and their management.	0.391	0.514
Recognize commonly used definitions of pain and be able to describe the implications of these definitions for the patient, e.g., central sensitization, allodynia, and hyperalgesia.	0.436	0.44

As hypothesized, the correlation between KNAP and part one of PUnCQ was significant (p = 0.006), with an r-value of 0.26 (95% CIs, 0.08-0.42). Similarly, the correlation between KNAP and part two of PUnCQ was significant (p < 0.001), with an r-value of 0.41 (95% CIs, 0.25-0.56).

## Discussion

This cross-cultural validity study investigated the structural and construct validity of the PUnCQ, which has the potential to assess an aspect of the clinical competence in pain management of Japanese physical therapists. The confirmatory factor analysis indicates that part one of the PUnCQ met all criteria for unidimensionality, indicating structural validity. However, doubts arose for part two regarding unidimensionality. Exploratory factor analysis for part two revealed a two-factor structure: a 14-item Factor 1 labeled “pain management” and a seven-item Factor 2 labeled “medication guidance and pain mechanism.” This finding may reflect that Japanese physical therapists do not receive pharmacology education. Although internal consistency for all items in part two was acceptable and the average of all items may be used for international comparisons, the average of both factors’ means may be reasonable when comparisons within the Japanese population are designed. Additionally, partial confirmation of the construct validity of PUnCQ was achieved.

Concerning PUnCQ scores, a study involving various professions, including students in Scottish physical therapy graduate programs and undergraduate nursing students (n = 2,238), reported a median of 4 for PUnCQ part one [11]. In contrast, a survey targeting undergraduate final-year physical therapy students in Scotland (n = 133) [10] reported that at least 50% of students selected the correct answer in almost all aspects. The mean score in this study for part one was 7.3, potentially aligning closely with the latter study’s data. However, items 3, 4, and 11 had a correct response rate of less than 40%, which may indicate a biomedical perspective on pain. These results may indicate the need to promote the education of Japanese physical therapists on the importance of the biopsychosocial perspective. For part two of the PUnCQ, the former previous study [11] reported average scores of 3.7 to 6.3, while the latter previous study [10] reported scores between 4 and 7. This study’s average score of 5.2 appears to approximate values from both previous studies. Although data suggest that the knowledge and understanding of pain among Japanese physical therapists may be lower than in advanced countries [26,27], this study includes a cohort of certified physical therapists in the McKenzie method, known for higher adherence to evidence-based practice [28], potentially contributing to the overall PUnCQ scores and resembling those in Scotland.

Regarding implications for physical therapy practice, this study enabled scoring PUnCQ, allowing us to move on to the final step of the questionnaire establishment process per the COSMIN. Thus, future research should verify the reliability and responsiveness of utilizing PUnCQ as a tool for assessing the effectiveness of educational interventions. Further, it is now possible to use the PUnCQ part one to identify relevant factors to enhance a certain competence in evidence-based clinical decision-making in relation to pain.

Several limitations exist in this study. First, the convenience sampling method introduces self-selection bias. However, since this is an anonymous survey, it is not assumed that only those who are confident in their pain management participated in this study, and therefore it is not believed that this limitation will significantly influence the results of this study. Second, the study used an adequate sample size according to COSMIN guidelines [14], but larger sample sizes would be desirable for a more accurate construct validity assessment. Third, the sample was limited to physical therapists, yet PUnCQ can be applied across various professions. Future research should consider larger multidisciplinary samples to assess the questionnaire’s validity. Fourth, the results of this study were obtained for Japanese physical therapists, and it is unclear whether similar internal structures are found among physical therapists in other countries. When using the PUnCQ in other countries, it would be necessary to confirm the internal structure, as in this study. Fifth, the clinical vignettes used in this study addressed disability related to whiplash injuries, and it is unclear whether the PUnCQ part 1 scores accurately reflect the competence of managing a variety of patients with pain, and further research is needed.

## Conclusions

This study confirmed the structural validity of the PUnCQ and partially supported its construct validity. While additional steps and further research are needed to establish the questionnaire according to COSMIN guidelines and to assess its applicability in other countries, the PUnCQ holds the potential for evaluating educational outcomes and the quality of Japanese physical therapists in the future.
